# A survey of current practices, attitudes and demands of anaesthesiologists regarding the depth of anaesthesia monitoring in China

**DOI:** 10.1186/s12871-021-01510-7

**Published:** 2021-11-23

**Authors:** Jian Zhan, Ting-Ting Yi, Zhuo-Xi Wu, Zong-Hong Long, Xiao-Hang Bao, Xu-Dong Xiao, Zhi-Yong Du, Ming-Jun Wang, Hong Li

**Affiliations:** 1 Department of Anaesthesiology, Second Affiliated Hospital of Army Medical University, Chongqing, 400037 China; 2 Department of Anaesthesiology, Affiliated Hospital of Southwest Medical University, Luzhou, 646000 Sichuan China; 3 Department of Anaesthesiology, Yongchuan Hospital, Chongqing Medical University, Chongqing, 402160 China; 4 Department of Anaesthesiology, The First Medical Center of Chinese PLA General Hospital, Beijing, 100853 China

**Keywords:** Depth of anaesthesia, Anaesthesiologists, Awareness, Analgesia, Artificial intelligence

## Abstract

**Background:**

In this study, we aimed to analyse survey data to explore two different hypotheses; and for this purpose, we distributed an online survey to Chinese anaesthesiologists. The hypothetical questions in this survey include: (1) Chinese anaesthesiologists mainly use the depth of anaesthesia (DoA) monitors to prevent intraoperative awareness and (2) the accuracy of these monitors is the most crucial performance factor during the clinical daily practice of Chinese anaesthesiologists.

**Methods:**

We collected and statistically analysed the response of a total of 12,750 anesthesiologists who were invited to participate in an anonymous online survey. The Chinese Society of Anaesthesiologists (CSA) trial group provided the email address of each anaesthesiologist, and the selection of respondents was random from the computerized system.

**Results:**

The overall response rate was 32.0% (4037 respondents). Only 9.1% (95% confidence interval, 8.2-10.0%) of the respondents routinely used DoA monitors. Academic respondents (91.5, 90.3-92.7%) most frequently used DoA monitoring to prevent awareness, whereas nonacademic respondents (88.8, 87.4-90.2%) most frequently used DoA monitoring to guide the delivery of anaesthetic agents. In total, the number of respondents who did not use a DoA monitor and whose patients experienced awareness (61.7, 57.8-65.6%) was significantly greater than those who used one or several DoA monitors (51.5, 49.8-53.2%). Overall, the crucial performance factor during DoA monitoring was considered by 61.9% (60.4-63.4%) of the respondents to be accuracy. However, most respondents (95.7, 95.1-96.3%) demanded improvements in the accuracy of the monitors for DoA monitoring. In addition, broad application in patients of all ages (86.3, 85.2-87.4%), analgesia monitoring (80.4, 79.2-81.6%), and all types of anaesthetic agents (75.6, 74.3-76.9%) was reported. In total, 65.0% (63.6-66.5%) of the respondents believed that DoA monitors should be combined with EEG and vital sign monitoring, and 53.7% (52.1-55.2%) believed that advanced DoA monitors should include artificial intelligence.

**Conclusions:**

Academic anaesthesiologists primarily use DoA monitoring to prevent awareness, whereas nonacademic anaesthesiologists use DoA monitoring to guide the delivery of anaesthetics. Anaesthesiologists demand high-accuracy DoA monitors incorporating EEG signals, multiple vital signs, and antinociceptive indicators. DoA monitors with artificial intelligence may represent a new direction for future research on DoA monitoring.

**Supplementary Information:**

The online version contains supplementary material available at 10.1186/s12871-021-01510-7.

## Background

Rapid advances in brain function monitoring technology have promoted the extensive application of electroencephalography (EEG) depth of anaesthesia (DoA) monitors in modern clinical practice. In the guidelines, DoA monitoring for brain function monitoring was published previously [1–3]. The guidelines mainly address the following three points. First, DoA monitoring is not currently routinely performed during general anaesthesia [2]. Second, the guidelines focus on the perioperative brain health of patients, particularly that of elderly patients. Elderly patients should undergo brain function monitoring, such as DoA monitoring, to reduce postoperative neurocognitive dysfunction [1]. Third, to reduce the risk of intraoperative awareness during general anaesthesia, the use of DoA monitors is recommended when patients are anaesthetized using total intravenous anaesthesia (TIVA) and muscle relaxants [2, 3]. Due to the limitations of DoA monitors based on current EEG technology, the role of these monitors in clinical practice is still controversial. There is ongoing improvement and continuous development of various aspects of the application of DoA monitors and their various uses [4, 5]. Currently, more than ten types of DoA monitors are available; these include the bispectral index (BIS) monitor (Aspect Medical Systems, Newton, MA, USA), Narcotrend (Monitor Technik, Bad Bramstedt, Germany), Entropy (GE Healthcare, Helsinki, Finland), and the cerebral state index (CSI) monitor (Danmeter A/S, Odense, Denmark). The first three of these are the most commonly used DoA monitors [6, 7]. These EEG-based monitors collect and process EEG data obtained using scalp electrodes. Several proprietary algorithms analyse the data and provide a dimensionless number that is intended to reflect DoA [8].

The scope of clinical application of DoA monitors continues to broaden. However, the use of DoA monitors in clinical practice needs to be widened, and a more positive attitude toward their daily routine use needs to be developed. Although intraoperative awareness is reduced when BIS monitoring is used, previous clinical studies did not find any difference in the incidence of awareness revealed by BIS monitoring values and end-tidal anaesthetic concentrations (ETAC) [9–11]. Additionally, compared with ETAC, BIS monitoring did not reduce the use of volatile anaesthetics [9], the patient’s recovery time, or the incidence of postoperative complications [12]. Other studies also showed that a cumulative BIS value below a threshold of 35 or 40 was not associated with increased postoperative mortality [13, 14].

Although DoA monitors are widely used in modern clinical practice, it has been reported that many factors potentially affect the usefulness of DoA monitors [4, 6, 15]. Current DoA monitors can only monitor hypnotic components; however, anaesthesia also involves an antinociceptive component [15]. Analgesia is defined as “insensibility to pain without loss of consciousness” (https://www.merriam-webster.com/dictionary/analgesia), whereas antinociception is defined as “the action or process of blocking the detection of a painful or injurious stimulus by sensory neurons” (https://www.merriam-webster.com/medical/antinociception). Therefore, the antinociceptive component includes analgesia and an anti-injury effect. Additionally, EEG signals are susceptible to age and to various pathophysiological conditions such as hypothermia, hypoglycaemia, acute cerebral hypoperfusion, and hypoxia and this can reduce the accuracy of DoA monitoring [16–19]. Furthermore, electromyography (EMG) interference and high cost are factors that affect the use of DoA monitors [6, 20].

A few surveys are available that provide information on how to safely and accurately use DoA monitors during anaesthesia practice as well as provide valuable information about the attitudes of anaesthesiologists regarding DoA monitors. These surveys were conducted in developed countries such as the United Kingdom and Australia [8, 21–23]. The surveys focused on awareness or on the primary driver for using DoA monitoring. Table 1 shows the characteristics of previous surveys. To the best of our knowledge, there are approximately 85,000 anaesthesiologists in China. However, these anaesthesiologists’ current practices, attitudes, and demands regarding DoA monitoring remain unclear. Therefore, we surveyed Chinese anaesthesiologists in an attempt to evaluate these variables and to understand the main purpose and functional requirements of Chinese anaesthesiologists regarding DoA monitoring. The present survey also aimed to provide clinical evidence for the application and functional improvement of DoA monitors.

**Table 1:** Characteristics of previous surveys

Authors	Journal	Publication year	Survey questions	Number of anaesthesiologists responding	Total number of anaesthesiologists	Response rate	Key results
Myles PS, et al. [22]	Anaesthesia	2003	22	186	220	85.0%	Many anaesthetists have had a patient who exhibited awareness under anaesthesia, but most rated awareness as only a moderate problem.
Lau K, et al. [21]	Eur J Anaesthesiol	2006	26	2170	4927	44.0%	Most anaesthetists accept that clinical signs are unreliable indicators of awareness; few believe that DoA monitors should be used for routine cases in the UK.
Ben-Menachem E, et al. [8]	Anesth Analg	2014	23	289	963	30.0%	The primary driver for the use of DoA monitoring in Australia is prevention of awareness.
Cheung YM, et al. [23]	BMC Anaesthesiol	2018	24	168	553	30.0%	Prevention of awareness is the most important reason to use DoA monitoring in children. The perceived lack of reliability of DoA monitoring in children is the most important reason not to use it.

## Methods

Approval to conduct the survey was obtained from the Medical Ethics Committee of the Second Affiliated Hospital of Army Medical University, Chongqing (ethics number: 2020-077-01).

During this study, we collected and statistically analysed the responses of a total of 12,750 anaesthesiologists to an anonymous online survey. The Chinese Society of Anaesthesiologists (CSA) trial group provided the email addresses of each anaesthesiologist, and the respondents were randomly selected using a computerized system. We developed a survey questionnaire similar to the previous surveys and guidelines [8, 24, 25]. The survey consisted of an introduction and 35 questions. The introduction included a brief project explanation of the project and encouraged participation. A small group of anaesthesiologists tested the survey questionnaire in advance to ensure that it was understandable by the potential respondents. Ten experienced anaesthesiologists at the Second Affiliated Hospital of Army Medical University gave their reports for their performance with the questionnaire. We performed a feedback analysis to test the face and content validity, and we revised and simplified four of the questions and avoid ambiguity. Before distributing the survey in its official form, the CSA trial group evaluated the questionnaire for representativeness and quality. The formal survey was then issued, and six full-time CSA scientific researchers managed the data.

The survey was posted on a questionnaire website (https://www.wenjuan.com/) and deployed using a WeChat QR code survey form. From July 16 to September 30, 2020, 12,750 certified clinical anaesthesiologists were randomly selected to participate in the survey. The study conductors sent the WeChat survey form to the anaesthesiologists with the assistance and support of the CSA trial group. Approximately 85,000 anaesthesiologists were CSA members in 2018–19; thus, the sampled population represented 15% of the total membership. The CSA is a national professional academic organization that represents most anaesthesiologists in China; as such, it is the training and supervisory body for anaesthesiologists. The anaesthesiologists who received the survey were randomly determined by using a computer program. The CSA trial group provided the list of the anaesthesiologists and their e-mail addresses. The sample size was calculated based on previous surveys. The overall response rate was approximately 30.0%, similar to the response rate in previous surveys [8, 22]. The sampled population represented 15.0% of the total membership (85,000 anaesthesiologists). The minimum sample size in this survey is 3825 survey forms (85,000 × 15.0% × 30.0% = 3825). All of the anesthesiologists who received the survey questionnaires did so via the internet through e-mail systems within the same two-weeks period. All the questionnaires were anonymous, and nonresponders did not provide any data.

The survey data were analysed using SPSS software (version 26.0; IBM Corp., Armonk, NY, USA). The data are expressed as proportions (rounded to 1 decimal place) with 95% confidence intervals. Statisticians evaluated the differences between categorical variables using the χ^2^ test.

## Results

Of the 12,750 WeChat QR code survey forms distributed, 4037 were completed. The overall response rate was 32.0%. The respondents were employed at 1641 hospitals across the country, including 1525 public hospitals and 116 private hospitals. Table 2 shows the demographic data of the respondents. The respondents’ demographic variables, which are representative of the membership of the CSA, included gender, age, title, geographic distribution, years of experience, and daily working hours [26]. In addition, there were four surveys regarding DoA monitoring from all over the world.

**Table 2:** Baseline characteristics of respondents

Characteristics of respondents —% (*N* = 4037 respondents)	
Gender	
Male	58.3(2352)
Female	41.7(1685)
Age (years)	
20–30	15.2(615)
30–40	44.6(1798)
40–50	27.9(1126)
> 50	12.3(498)
Academic degree	
Bachelor	65.5(2643)
Master	27.6(1116)
Doctor	6.9(278)
Job title	
Junior	24.6(993)
Intermediate	38.3(1547)
Deputy senior	25.7(1035)
Senior	11.4(462)
Region	
Eastern region	20.4(823)
Western region	26.9(1085)
Southern region	17.2(694)
Northern region	19.1(770)
Central region	16.5(665)
Years as a practising anaesthesiologist	
0–5 years	15.4(621)
5–9 years	22.3(900)
10–19 years	33.1(1335)
≥ 20 years	29.2(1181)
Average clinical work hours per day	
0-8 h	19.0(767)
9-11 h	71.6(2892)
≥ 12 h	9.4(378)

Among the several monitors commonly used for DoA monitoring, the BIS monitor was used by 81.0% (79.8-82.2%) of the respondents. Most of the respondents (68.5, 67.1-69.9%) believed that the BIS monitor is the most accurate instrument available for DoA monitoring, followed by the Narcotrend monitor (13.1, 12.0-14.1%). Regarding the most valuable indicator on DoA monitors, most of the respondents (65.4, 64.0-66.9%) believed that number, EEG trace, and burst suppression ratio are equally important; the remaining respondents specified number (28.3, 26.9-29.7%), EEG trace (5.5, 4.8-6.2%) or burst suppression ratio (0.8, 0.5-1.0%) as the most valuable indicator.

A large proportion of the respondents (81.0, 79.8-82.2%) agreed or strongly agreed that DoA monitoring should be mandatory during TIVA with muscle relaxants. In comparison, 71.4% (70.0-72.8%) of the respondents agreed or strongly agreed that DoA monitoring should be mandatory during TIVA without muscle relaxants.

In individual practice, DoA monitoring was routinely used by only 9.1% (8.2-10.0%) of the respondents; it was used in more than one-third of cases by 28.8% (27.5-30.3%), in less than one third of cases by 49.1% (47.5-50.6%), and never by 22.1% (20.8-23.4%). Many crucial factors influenced the use of DoA monitors. Poor anti-interference ability (56.0, 54.5-57.6%) and inability to bill insurance or high cost (55.5, 54.0-57.0%) were the main influencing factors, followed by limited accuracy (47.9, 46.3-49.4%) and inability to monitor analgesia (35.7, 34.2-37.1%).

Most of the respondents (96.5, 95.9-97.0%) believed that currently available DoA monitors are effective for DoA monitoring, and only 0.2% (0.1-0.4%) of the respondents thought they were invalid. Among the respondents, 85.2% (84.1-86.3%) reported that DoA monitoring could also reduce the use of anaesthetics. In clinical practice, anaesthesiologists can determine DoA using multiple monitoring methods combined with their own experience. Of the survey respondents, 65.9% (64.4-67.4%) of the respondents assessed DoA based on the dosage of anaesthetics and on vital signs. In contrast, only 21.7% (20.4-23.0%) did so using a DoA monitor, 10.1% (9.2-11.0%) did so using only vital signs, and 2.3% (1.8-2.8%) did so using ETAC.

The majority of the respondents (81.7, 80.5-82.9%) strongly agreed or agreed that prolonged low DoA readings (< 40) are associated with adverse outcomes. Additionally, most of the respondents (96.6, 96.0-97.1%) agreed or strongly agreed to deliver fewer anaesthetics with prolonged lower DoA readings (≤ 35). In comparison, 95.2% (94.6-95.9%) of the respondents agreed or strongly agreed to increase anaesthetic delivery with prolonged elevated DoA readings (≥ 65). The influence of low DoA readings was more significant than that of high DoA readings (*P* = 0.002, χ^2^ = 9.160).

Additionally, regarding the population for which DoA monitoring is applicable, approximately 91.9% (91.1-92.7%) of the respondents reported that elderly patients are most in need of DoA monitoring. Surprisingly, compared with the proportion of respondents who thought that such monitoring is suitable for youths (63.2, 61.7-64.7%; *P* < 0.001) or young children and infants (53.8, 52.2-55.3%; *P* < 0.001), the proportion (71.2, 69.8-72.6%) of the respondents who considered DoA monitoring suitable for adults was higher.

We performed a stratified analysis of the respondents regarding the topic of the purposes for using a DoA monitor; the results of that analysis are presented in Table 3. Surprisingly, more than half of the respondents (53.0, 51.5-54.5%) reported having had a case of awareness in individual practice. In contrast, 36.0% (34.6-37.5%) of the respondents had not had a case of awareness, and 10.9% (10.0-11.9%) did not know whether a case of awareness had occurred in their practice. Furthermore, most of the respondents (92.5, 91.7-93.3%) strongly agreed or agreed that DoA monitors can prevent awareness, and only 3.1% (2.5-3.6%) strongly disagreed or disagreed with this statement. Moreover, 68.5% (67.1-69.9%) of the respondents considered DoA monitoring more effective than ETAC in preventing awareness, 8.4% (7.5-9.3%) thought there was no difference, and 5.1% (4.4-5.8%) considered that DoA monitoring was inferior to ETAC monitoring.

**Table 3:** Stratified analysis of the respondents regarding the purposes of using a DoA monitor

	Preventing awareness	Guiding the delivery of anaesthetics	Reducing recovery time	Avoiding deep anaesthesia	Preventing side effects of anaesthetics	Determining the cause of drastic changes in hemodynamics
All respondents (*N* = 4037)	89.9% (89.0-90.8%)	88.0% (87.0-89.0%)	75.2% (73.8-76.5%)	87.5% (86.5-88.6%)	48.1% (46.6-49.7%)	62.1% (60.6-63.6%)
Academic respondents (*n* = 2066)	91.5% (90.3-92.7%)	87.2% (85.8-88.6%)	73.3% (71.4-75.2%)	87.6% (86.2-89.0%)	45.4% (43.3-47.6%)	59.3% (57.2-61.4%)
Nonacademic respondent (*n* = 1971)	88.2% (86.8-89.6%)	88.8% (87.4-90.2%)	77.2% (75.4-79.1%)	87.5% (86.0-89.0%)	51.0% (48.8-53.2%)	65.0% (62.9-67.1%)

*DoA* Depth of anaesthesia

In a stratified analysis, we asked the anaesthesiologists whether they used a DoA monitor and what type of monitor they preferred. The number of respondents who had not used a DoA monitor and had had a case of experienced awareness (61.7, 57.8-65.6%) was greater than the number who had used one or several DoA monitors and had had a case of awareness (51.5%, 49.8-53.2%). The percentage of respondents who had not had a case of experienced awareness was also lower (27.2% vs. 37.6%). According to the stratified analysis of the main methods used by the respondents to assess the DoA, those who relied only on changes in vital signs to determine the DoA had higher percentages of cases of awareness during the operation than those who used the other three methods as shown in Table 4.

**Table 4:** Stratified analysis of the relationship between the use of different DoA monitors and the main means to assess DoA by the respondents and the occurrence of awareness

	Have you experienced a case of awareness in the past?		
	Had a case of awareness	Did not have a case of awareness	Do not know
All respondents (*N* = 4037)	53.0% (51.5-54.6%)	36.0% (34.6-37.5%)	11.0% (10.0-11.9%)
Respondents who had used a DoA monitor (*n* = 3434)	51.5% (49.8-53.2%)	37.6% (36.0-39.2%)	10.9% (9.9-11.9%)
BIS (*n* = 3270)	51.6% (49.9-53.3%)	37.7% (36.0-39.4%)	10.7% (9.6-11.8%)
Entropy (*n* = 257)	50.2% (44.1-56.3%)	37.4% (31.5-43.3%)	12.5% (8.5-16.5%)
Narcotrend (*n* = 838)	50.5% (47.1-53.9%)	36.9% (33.6-40.2%)	12.6% (10.4-14.9%)
AEP (*n* = 154)	58.4% (50.6-66.2%)	34.4% (26.9-41.9%)	7.1% (3.0-11.2%)
CSI (*n* = 436)	50.5% (45.8-55.2%)	37.2% (32.7-41.7%)	12.4% (9.3-15.5%)
PSI (*n* = 116)	52.6% (43.5-61.7%)	32.8% (24.3-41.3%)	14.7% (8.3-21.1%)
Respondents who had never used a DoA monitor (*n* = 603)	61.7% (57.8-65.6%)	27.2% (23.7-30.8%)	11.1% (8.6-13.6%)
The main means to assess DoA			
Only vital signs (*n* = 408)	59.3% (54.5-64.1%)	31.4% (26.9-35.9%)	9.3% (6.5-12.1%)
ETAC (*n* = 93)	45.2% (35.1-55.3%)	40.9% (30.9-50.9%)	14% (7.0-21.1%)
DoA monitor (*n* = 876)	53.0% (49.7-56.3%)	39.4% (36.2-42.6%)	7.6% (5.9-9.4%)
Dosage of anaesthetics and vital signs (*n* = 2660)	52.3% (50.4-54.2%)	35.5% (33.7-37.3%)	12.2% (11.0-13.4%)

*DoA* Depth of anaesthesia, *BIS* Bispectral index, *AEP* Auditory evoked potentials, *CSI* Cerebral state index, *PSI* Patient state index, *ETAC* End-tidal anaesthetic concentration

Regarding the performance demands of DoA monitoring, 61.9% (60.4-63.4%) of the respondents considered accuracy the most crucial factor related to performance, while applicability (20.0, 18.8-21.3%), stability (15.9, 14.7-17.0%), and cost-effectiveness (2.2, 1.8-2.7%) were also valued.

Based on the current DoA monitors used, the respondents believed that the DoA monitoring functions that required further improvement are as follows: accuracy (95.7, 95.1-96.3%), suitability for patients of all ages (86.3, 85.2-87.4%), analgesia (80.4, 79.2-81.6%), and suitability for use with all anaesthetics (75.6, 74.3-76.9%). Additionally, most (65.0, 63.6-66.5%) thought DoA monitors should combine EEG and vital sign monitoring. More than half of the respondents (53.7, 52.1-55.2%) believed that advanced DoA monitors should use artificial intelligence. Regarding the application of DoA monitoring and whether DoA monitoring has become as crucial as electrocardiogram (ECG) monitoring in clinical practice, 90.1% (89.1-91.0%) of the respondents reported a significant difference between the importance of DoA monitoring and that of ECG monitoring.

## Discussion

DoA monitoring is crucial for anaesthesia management; however, the use of DoA monitors is not currently widespread. Academic anaesthesiologists mainly use DoA monitoring to prevent awareness, while nonacademic anaesthesiologists mainly use DoA monitoring to guide the delivery of anaesthetics. Poor anti-interference ability, inability to bill insurance or high cost are the main factors that influence the application of DoA monitors. The accuracy of DoA monitors was considered the most crucial performance factor and was requested by most respondents. The respondents also indicated that, ideally, DoA monitoring should be suitable for patients of all ages and for use with all anaesthetics. The availability of DoA monitoring that includes analgesia monitoring and artificial intelligence is also crucial.

The respondents report that the BIS monitor is the most commonly used (81.0%) DoA monitor. This result is consistent with previous surveys [8, 23]. These surveys indicate that the BIS monitor has the highest use and the greatest acceptance by anaesthesiologists in these countries in which the surveys were conducted. Unlike a previous survey conducted in Europe in 2018 that focused on the use, applicability and reliability of DoA monitors in children, the current survey focused on the practices, attitudes and demands of anaesthesiologists regarding DoA monitoring in patients of all ages.

Although DoA monitors are currently used in anaesthesia departments in most Chinese hospitals, more than one-fifth of the respondents had never used DoA monitoring. The proportion of respondents who reported that they routinely used DoA monitors was higher in this study than in a survey conducted in Australia in 2014 (9.1% vs. 1.0%) [8]. However, in order to prevent awareness, the proportion of routine use of DoA monitors in the UK in 2006 (22.0%) [21] and in Australia in 2003 (53.0%) [22] were higher than that in China. A possible explanation for this may be that the increasing availability of DoA monitoring and its usefulness in preventing awareness have promoted its use. These reasons were also cited in previous studies that published survey results [4, 6, 15]. We consider that the decrease in the incidence of intraoperative awareness has led to a decrease in the percentage of anaesthesiologists who use DoA monitors in Australia. The main purpose reported by respondents in these two studies in using DoA monitoring was to prevent intraoperative awareness. Approximately half (52.0%) of the respondents surveyed in Australia in 2003 had experienced a patient with awareness [22]. In Australia in 2014, 30.0 % of respondents reported having had a case of awareness in their practice [8]. The use of DoA monitoring is also related to the choice of subjects for monitoring by anaesthesiologists. Whether DoA monitoring should be used in all patients undergoing general anaesthesia or only in those at high risk of awareness remains debatable [16].

Interestingly, academic anaesthesiologists mainly used DoA monitoring to prevent awareness, while nonacademic anaesthesiologists mainly used DoA monitoring to guide the delivery of anaesthetics. This survey also found that current DoA monitors have known limitations, including limited accuracy [4], poor anti-interference ability [4], inability to monitor analgesia [15], and lack of unsuitability for patients of all ages [6]. A failure to bill insurance or high cost is an essential factor that affects the routine use of DoA monitoring [9, 20]. Overall, our findings suggest that the cost-effectiveness of DoA monitoring in reducing the likelihood of intraoperative awareness or the delivery of anaesthetics is limited.

The type of DoA monitor used had no perceived effect on the rate of awareness experienced by the respondents. This finding suggests that no perceived difference in the prevention of awareness was noted among different types of DoA monitors. Furthermore, more than two-thirds of the respondents believed that DoA monitoring was more effective than ETAC in preventing awareness. This finding is consistent with the results of the 2014 Australian survey [8]. However, Avidan et al. reported no difference between DoA monitoring and ETAC in preventing awareness [11]. The reason for this may be that the behaviour of anaesthesiologists does not always reflect their knowledge of the evidence. Therefore, awareness of the difference between practice and evidence may encourage anaesthesiologists to reflect on their own behaviours and guide evidence into clinical practice.

Most of the respondents (91.9%) thought that DoA monitoring should be used with elderly patients, and a few believed that DoA monitoring should also be used with infants and young children. However, the incidence of awareness in adults is 0.1-0.2% [8], and that in children is 0.7% [27], suggesting that DoA monitoring should be used more often in children than adults. The difference between the results of this survey and the findings reported in previous studies may be attributed to the facts that current DoA monitors are not suitable for use with infants and young children [28] and that elderly patients are more likely to experience complications such as postoperative delirium [29]. Furthermore, most respondents thought that accuracy was the most crucial performance in DoA monitoring. However, previously, some anaesthesiologists considered that DoA monitors could accurately guide the physician to perform anaesthesia management. This could also suggest a response of the anaesthesiologist to DoA monitoring [30].

With the development of EEG research and technology, the demands of anaesthesiologists regarding DoA monitoring are changing. Currently available DoA monitors can monitor only the depth of sedation [4]. However, the proprietary algorithms used by DoA monitors are accurate approximately 65.0 to 85.0% of the time [31]. Our results also suggest that current DoA monitors have limited accuracy and indicate that availability of DoA monitors with high accuracy is the primary demand of the respondents. Most respondents thought that DoA monitors should combine EEG and vital sign monitoring, given that combined monitoring may reflect differences in DoA more precisely [32]. Although analgesia is essential for general anaesthesia, current DoA monitors cannot monitor analgesia [15]. Our results indicate that analgesia monitoring is also a crucial demand of most (80.4%) respondents. Therefore, future DoA monitoring should integrate EEG, other exogenous information, and antinociceptive indices in predicting DoA [31].

Future research on DoA monitoring should focus on artificial intelligence. More than half of the respondents thought ideal DoA monitoring should include artificial intelligence, and artificial intelligence is well suited for use in the analysis of complex data, including EEG signals [5]. The use of artificial intelligence combined with EEG and measurement of multiple vital signs to predict DoA is more accurate than the BIS monitor [33, 34]. Prediction of the level of sedation based on numerous EEG features in combination with an artificial intelligence algorithm was shown to be independent of the anaesthetic drugs used [35], indicating that artificial intelligence-based DoA monitors are feasible for use in scenarios involving a wide variety of anaesthetics. Furthermore, EEGs of patients under general anaesthesia show changes related to the patient’s age, and current DoA monitors cannot explain these age-related changes [4, 36, 37]. Therefore, future DoA monitoring must measure and analyse the EEG features of patients of different ages during the use of different anaesthetics to achieve individualized accurate prediction, and future anaesthesia practice is likely to include routine monitoring of the brain [38].

Although current guidelines effectively guide the reasonable use of DoA monitors, the guidelines do not exclusively encourage the use of these monitors. First, DoA monitoring is currently not routinely conducted for patients under general anaesthesia [2]. This may be related to the limitations of current DoA monitors, as these limitations affect their widespread use [4, 15]. Second, the guidelines focus on the perioperative brain health of patients, particularly elderly patients. A possible reason for this is that elderly persons are more prone to postoperative neurocognitive dysfunction, and DoA monitoring can reduce its occurrence [1]. Furthermore, DoA monitoring is an essential method of monitoring brain function, and future anaesthesia practice is likely to include routine monitoring of the brain [38]. Finally, the use of DoA monitors during TIVA and during the administration of muscle relaxants is recommended. Previous studies have shown that DoA monitoring can reduce the risk of intraoperative awareness during TIVA and when muscle relaxants are used [39, 40]. Additionally, the daily practice of the guidelines in the survey reflects DoA monitoring during anaesthesia practice in China. Several previous studies showed reflections from other countries, including the United Kingdom and Australia [8, 21, 22].

This survey has some limitations. First, the sample size was limited (the survey population accounted for 15.0% of all anaesthesiologists in China), with a response rate of 32.0%. However, this response rate is a typical for online surveys [8, 25] and may introduce the possibility of nonresponse bias. However, the demographic characteristics of the respondents to this survey, including sex, age, geographic distribution, professional titles, years of experience, and daily working hours, closely reflect the membership of the CSA [26], supporting the representativeness of this survey. Additionally, the large sample size is a strength of this study. Among recently conducted surveys regarding the use of DoA monitors, this survey has the largest sample size (4037 respondents among 12,750 surveyed anaesthesiologists). Second, although the selection of respondents was random, their responses may be nonrandom, and there may potentially be response bias. We cannot confirm the validity of the responses because the inference of these surveys may be subjective. The respondents’ representativeness of the total population may be more important than selection bias. However, we checked the eligibility and the integrity of the data for each individual survey, and no data were lost. There is a possibility of nonresponse bias in our study because the way in which the respondents were chosen may select for respondents with strong attitudes towards this subject. Another limitation of this study is that most respondents (95.9%, *N* = 3873) were employed at public hospitals; only 4.1% (*N* = 164) were employed at private hospitals. This may be related to the structure of the medical system in China, which is based mainly on public hospitals. Finally, this survey reflects only Chinese anaesthesiologists’ practices, attitudes and demands regarding DoA monitoring, and the findings cannot be generalized to other countries or regions given international variations.

## Conclusions

We conducted the first survey performed to date of the current practices, attitudes and demands of anaesthesiologists in China regarding DoA monitoring. Academic anaesthesiologists primarily use DoA monitoring to prevent awareness, while nonacademic anaesthesiologists primarily use DoA monitoring to guide the delivery of anaesthetics. Anaesthesiologists demand high-accuracy DoA monitors that take into account EEG signals, multiple vital signs, and antinociceptive indicators. DoA monitors that use artificial intelligence may represent a new direction for future research on DoA monitoring.

## Supplementary Information

**Additional file 1.** The survey questionnaire.

## Data Availability

The datasets are not publicly available but are available from the corresponding author on reasonable request.

## Abbreviations

DoA: Depth of anaesthesia
EEG: Electroencephalogram
BIS: Bispectral index
CSI: Cerebral state index
EMG: Electromyography
ETAC: End-tidal anaesthetic concentration
CSA: Chinese Society of Anaesthesiologists
TIVA: Total intravenous anaesthesia
ECG: Electrocardiogram

## References

[1] Mahanna-Gabrielli E, Schenning KJ, Eriksson LI, Browndyke JN, Wright CB, Culley DJ (2019). State of the clinical science of perioperative brain health: report from the American Society of Anaesthesiologists Brain Health Initiative Summit 2018. Br J Anaesth.

[2] Checketts MR, Alladi R, Ferguson K, Gemmell L, Handy JM, Klein AA (2016). Recommendations for standards of monitoring during anaesthesia and recovery 2015: Association of Anaesthetists of Great Britain and Ireland. Anaesthesia.

[3] Aldecoa C, Bettelli G, Bilotta F, Sanders RD, Audisio R, Borozdina A (2017). European Society of Anaesthesiology evidence-based and consensus-based guideline on postoperative delirium. Eur J Anaesthesiol.

[4] Shander A, Lobel GP, Mathews DM (2018). Brain monitoring and the depth of anaesthesia: another goldilocks dilemma. Anesth Analg.

[5] Hashimoto DA, Witkowski E, Gao L, Meireles O, Rosman G (2020). Artificial intelligence in anesthesiology: current techniques, clinical applications, and limitations. Anaesthesiology.

[6] Fahy BG, Chau DF (2018). The technology of processed electroencephalogram monitoring devices for assessment of depth of anaesthesia. Anesth Analg.

[7] Hajat Z, Ahmad N, Andrzejowski J (2017). The role and limitations of EEG-based depth of anaesthesia monitoring in theatres and intensive care. Anaesthesia.

[8] Ben-Menachem E, Zalcberg D (2014). Depth of anaesthesia monitoring: a survey of attitudes and usage patterns among Australian Anaesthesiologists. Anesth Analg.

[9] Avidan MS, Zhang L, Burnside BA, Finkel KJ, Searleman AC, Selvidge JA (2008). Anaesthesia awareness and the bispectral index. N Engl J Med.

[10] Myles PS, Leslie K, McNeil J, Forbes A, Chan MT (2004). Bispectral index monitoring to prevent awareness during anaesthesia: the B-Aware randomised controlled trial. Lancet.

[11] Avidan MS, Jacobsohn E, Glick D, Burnside BA, Zhang L, Villafranca A (2011). Prevention of intraoperative awareness in a high-risk surgical population. N Engl J Med.

[12] Fritz BA, Rao P, Mashour GA, Abdallah AB, Burnside BA, Jacobsohn E (2013). Postoperative recovery with bispectral index versus anesthetic concentration-guided protocols. Anesthesiology.

[13] Kertai MD, Palanca BJ, Pal N, Burnside BA, Zhang L, Sadiq F (2011). Bispectral index monitoring, duration of bispectral index below 45, patient risk factors, and intermediate-term mortality after noncardiac surgery in the B-Unaware Trial. Anaesthesiology.

[14] Short TG, Campbell D, Frampton C, Chan MTV, Myles PS, Corcoran TB (2019). Anaesthetic depth and complications after major surgery: an international, randomised controlled trial. Lancet.

[15] Pilge S, Schneider G (2015). BIS and state entropy of the EEG - comparing apples and oranges. Br J Anaesth.

[16] Bruhn J, Myles PS, Sneyd R, Struys MM (2006). Depth of anaesthesia monitoring: what’s available, what’s validated and what’s next?. Br J Anaesth.

[17] Voss L, Sleigh J (2007). Monitoring consciousness: the current status of EEG-based depth of anaesthesia monitors. Best Pract Res Clin Anaesthesiol.

[18] Wu L, Zhao H, Weng H, Ma D (2019). Lasting effects of general anesthetics on the brain in the young and elderly: “mixed picture” of neurotoxicity, neuroprotection and cognitive impairment. J Anesth.

[19] Dahaba AA (2005). Different conditions that could result in the bispectral index indicating an incorrect hypnotic state. Anesth Analg.

[20] Shepherd J, Jones J, Frampton G, Bryant J, Baxter L, Cooper K (2013). Clinical effectiveness and cost-effectiveness of depth of anaesthesia monitoring (E-Entropy, Bispectral Index and Narcotrend): a systematic review and economic evaluation. Health Technol Assess.

[21] Lau K, Matta B, Menon DK, Absalom AR (2006). Attitudes of anaesthetists to awareness and depth of anaesthesia monitoring in the UK. Eur J Anaesthesiol.

[22] Myles PS, Symons JA, Leslie K (2003). Anaesthetists’ attitudes towards awareness and depth-of-anaesthesia monitoring. Anaesthesia.

[23] Cheung YM, Scoones G, Stolker RJ, Weber F (2018). Use, applicability and reliability of depth of hypnosis monitors in children - a survey among members of the European Society for Paediatric Anaesthesiology. BMC Anesthesiol.

[24] Burmeister LF (2003). Principles of successful sample surveys. Anesthesiology.

[25] Jones D, Story D, Clavisi O, Jones R, Peyton P (2006). An introductory guide to survey research in anaesthesia. Anaesth Intensive Care.

[26] Huang Y-G, Deng X-M (2019). Chinese medical development series research report-advances in anesthesiology.

[27] Davidson AJ, Smith KR, Blussé van Oud-Alblas HJ, Lopez U, Malviya S, Bannister CF (2011). Awareness in children: a secondary analysis of five cohort studies. Anaesthesia.

[28] Davidson AJ (2007). Monitoring the anaesthetic depth in children - an update. Curr Opin Anaesthesiol.

[29] Hughes CG, Boncyk CS, Culley DJ, Fleisher LA, Leung JM, McDonagh DL (2020). American Society for Enhanced Recovery and Perioperative Quality Initiative Joint Consensus Statement on postoperative delirium prevention. Anesth Analg.

[30] Escallier KE, Nadelson MR, Zhou D, Avidan MS (2014). Monitoring the brain: processed electroencephalogram and peri-operative outcomes. Anaesthesia.

[31] Sleigh J (2014). No monitor is an island: depth of anaesthesia involves the whole patient. Anesthesiology.

[32] Schneider G, Jordan D, Schwarz G, Bischoff P, Kalkman CJ, Kuppe H (2014). Monitoring depth of anaesthesia utilizing a combination of electroencephalographic and standard measures. Anesthesiology.

[33] Sadrawi M, Fan SZ, Abbod MF, Jen KK, Shieh JS (2015). Computational depth of anaesthesia via multiple vital signs based on artificial neural networks. Biomed Res Int.

[34] Shalbaf A, Saffar M, Sleigh JW, Shalbaf R (2018). Monitoring the depth of anaesthesia using a new adaptive neurofuzzy system. IEEE J Biomed Health Inform.

[35] Ramaswamy SM, Kuizenga MH, Weerink MAS, Vereecke HEM, Struys MMRF, Nagaraj SB (2019). Novel drug-independent sedation level estimation based on machine learning of quantitative frontal electroencephalogram features in healthy volunteers. Br J Anaesth.

[36] Purdon PL, Pavone KJ, Akeju O, Smith AC, Sampson AL, Lee J (2015). The ageing brain: age-dependent changes in the electroencephalogram during propofol and sevoflurane general anaesthesia. Br J Anaesth.

[37] Ni K, Cooter M, Gupta DK, Thomas J, Hopkins TJ, Miller TE (2019). Paradox of age: older patients receive higher age-adjusted minimum alveolar concentration fractions of volatile anaesthetics yet display higher bispectral index values. Br J Anaesth.

[38] Avidan MS, Mashour GA (2013). Prevention of intraoperative awareness with explicit recall: making sense of the evidence. Anesthesiology.

[39] Gao WW, He YH, Liu L, Yuan Q, Wang YF, Zhao B (2018). BIS monitoring on intraoperative awareness: a meta-analysis. Curr Med Sci.

[40] Zhang C, Xu L, Ma YQ, Sun YX, Li YH, Zhang L (2011). Bispectral index monitoring prevent awareness during total intravenous anesthesia: a prospective, randomized, double-blinded, multi-center controlled trial. Chin Med J.

