# Supplementary material for: A survey of current practices, attitudes and demands of anaesthesiologists regarding the depth of anaesthesia monitoring in China
Source: BMC Anesthesiol. 2021 Nov 23;21:294. doi: 10.1186/s12871-021-01510-7 (PMC8609812; doi:10.1186/s12871-021-01510-7)
Supplement: Supplementary file 1 — Additional file 1. The survey questionnaire. [file 12871_2021_1510_MOESM1_ESM.docx]

**A questionnaire survey for depth of anaesthesia monitoring**

Dear colleagues,

In order to successfully complete the National Key R&D Program

of the Ministry of Science and Technology of China, "Evaluation on instruments of perioperative vital sign monitoring based on Internet of Things Technology",we sincerely invite you to participate in this survey regarding the views of anaesthesiologists on depth of anaesthesia (DoA) monitoring and its application in China. This questionnaire will be one of the main data sources of the Ministry of Science and Technology project. The data and information obtained are strictly confidential and will only be used for project research and reference to the superior authorities, and will not be used for any profit-making activities or commercial gain. Thank you very much for your cooperation!

Please fill in the relevant content in the following horizontal line or box with "tick".

1. What is your gender?

□Male; □Female

2. What is your age(years)?

□20-30; □30-40; □40-50; □>50

3. What isyour academic degree?

□Bachelor; □Master; □Doctor

4. What is your job title?

□Senior;□Deputy senior; □Intermediate; □Junior

5. How many years as a practising anaesthesiologist do you have?

□< 5 years;□5-9 years; □10-19 years;□ ≥ 20 years

6. How many clinical work hours per day do you have?

□<8 hours; □9-11 hours; □≥12 hours

7. What is the name of the hospital where you work?

___________________________

8. What is the level of the hospital where you work?

□ Class A Grade Ⅲ teaching; □ Class A Grade Ⅲ non-teaching; □ Class B(or C) Grade Ⅲ; □Class A Grade II; □ Class B Grade II(or below)

9. What is the ownership of the hospital where you work?

□Public hospital;□Private hospital

10. Are you familiar with using of DoA monitors?

□Very familiar; □Familiar; □Unfamiliar

11. Do you think a DoA monitor is effective for DoA monitoring?

□Very effective; □Little effective; □Invalid; □Unknown

12. What is the main means to evaluate DoA in your daily work?

□Only vital signs; □ETAC; □A DoA monitor; □Dosage of anaesthetics and vital signs

13. Are you satisfied with the DoA achieved in clinical practice?

□Very satisfied; □Satisfied;□Dissatisfied; □Very dissatisfied

14.What performance do you value most when choosing a DoA monitor?

□Accuracy; □Stability; □Cost-effectiveness; □Applicability

15. Which brand do you prefer when it comes to a DoA monitor?

□Domestic brands; □Import brands;□Both

16.Which kind of DoA monitor have you used before? (multiple options)

□BIS; □Entropy; □Narcotrend; □AEP; □CSI; □PSI; □Never used

17. Which kind of DoA monitor do you think is the most accurate for DoA monitoring?

□BIS; □Entropy; □Narcotrend; □AEP; □CSI; □PSI;□Others types

18.What do you think is the most valuable indicator of a DoA monitor?

□The number; □EEG trace; □Burst suppression ratio;□The three indicators are equally important

19.What do you think is the proportion of cases using a DoA monitor in clinical practice?

□Never; □< 1/3; □1/3~2/3; □> 2/3; □Always

20.What do you think are the main purposes of using a DoA monitor in clinical practice? (multiple options)

□Preventing awareness; □Guiding the delivery of anaesthetics; □Reducing recovery time; □Avoiding deep anaesthesia;□Preventing side effects of anaesthetics; □Determining the cause of drastic changes in hemodynamics

21.Which anaesthesia methods do you think is suitable for DoA monitoring in patients? (multiple options)

□General anaesthesia with tracheal intubation; □General anaesthesia with spontaneous breathing; □Local anaesthesia with sedation; □Intravenous anaesthesia for painless diagnosis and treatment; □All general anaesthesia

22.Do you agree that DoA monitors can prevent intraoperative awareness?

□Strongly agree; □Agree; □Don't know; □Disagree; □Strongly disagree

23.Have you ever had a case of awareness in individual practice?

□Yes;□No;□Don't know

24.Do you agree that inhaled anaesthetics are useful in preventing intraoperative awareness?

□Strongly agree; □Agree; □Don't know; □Disagree; □Strongly disagree

25.Compared with end-tidal anaesthetic concentration (ETAC), do you think that DoA monitoring is more effective in preventing intraoperative awareness?

□More effective; □No difference; □Inferior;□ Don't know

26.Do you agree that DoA monitoring can reduce the use of anaesthetics during anaesthesia?

□Agree; □Disagree;□Don't know

27.Do you agree that DoA monitoring should be mandatory during total intravenous anaesthesia (TIVA)with muscle relaxants?

□Strongly agree; □Agree; □Don't know; □Disagree; □Strongly disagree

28.Do you agree that DoA monitoring should be mandatory during total intravenous anaesthesia(TIVA)without muscle relaxants?

□Strongly agree; □Agree; □Don't know; □Disagree; □Strongly disagree

29.Do you agree that prolonged low DoA readings (< 40) are associated with adverse outcomes?

□Strongly agree; □Agree; □Don't know; □Disagree; □Strongly disagree

30.Will you decrease delivered anaesthetic if prolonged DoA readings are less than or equal to 35during operation?

□Strong disagreement; □Disagree; □Agree; □Strongly agree

31.Will you increase delivered anaesthetic if prolonged DoA readings are more than or equal to 65 during operation?

□Strong disagreement; □Disagree; □Agree; □Strongly agree

32. What do you think are the main factors that influence the application of DoA monitors? (multiple choices)

□Limited accuracy;□Limited sensitivity;□Unintuitive monitoring indicators or difficult to understand; □Little guidance for anesthesia management□Inability to monitor analgesia; □Poor anti-interference ability; □Complex operation; □Inability to bill insurance or high cost; □Others

33.Which kind of population do you think is suitable for DoA monitoring? (multiple choices)

□Young children and infants; □Youth; □Adults; □The elderly

34.How far do you think there is still to go before DoA monitoring becomes as important as ECG monitoring in clinical practice?

□A significant gap;□A little gap;□No gap;□The former has surpassed the latter

35.What kind of performance do you think an ideal DoA monitor should have? (multiple choices)

□Accuracy;□Suitability for patients of all ages;□Suitability for all anaesthetics; □Analgesia monitoring; □Cognitive function monitoring; □Artificial intelligence;□Strong anti-interference ability;□Simple and convenient operation; □Combined EEG and vital sign monitoring; □Others
